# Predictive model of sperm DNA fragmentation in infertile men based on lifestyle factors

**DOI:** 10.3389/fendo.2025.1675168

**Published:** 2025-10-27

**Authors:** Mengjia Pan, You Zhang, Ningxin Qin, Yan Xu, Sang Ni, Wei Chen, Xin Huang, Ke Wang

**Affiliations:** ^1^ Shanghai Key Laboratory of Maternal Fetal Medicine, Shanghai Institute of Maternal-Fetal Medicine and Gynecologic Oncology, Shanghai First Maternity and Infant Hospital, School of Medicine, Tongji University, Shanghai, China; ^2^ School of Medicine, Sanda University, Shanghai, China; ^3^ Center of Reproductive Medicine, Xinhua Hospital Affiliated Shanghai Jiao Tong University, School of Medicine, Shanghai, China

**Keywords:** infertile men, sperm DNA fragmentation rate, predictive model, risk factors, nomogram

## Abstract

**Objective:**

This study aimed to investigate the influence of lifestyle factors on the sperm DNA fragmentation index (DFI) in infertile men, and to develop and validate a predictive model for identifying individuals at risk of abnormal DFI.

**Methods:**

A total of 746 infertile men who underwent intracytoplasmic sperm injection and embryo transfer (ICSI-ET) at the Obstetrics and Gynecology Hospital affiliated with Tongji University from June 2023 to December 2024 were included as the training cohort, while 308 infertile men treated at Xinhua Hospital affiliated with Shanghai Jiao Tong University School of Medicine from January to June 2024 served as the external validation cohort. Data were collected using structured questionnaires, including general demographic information, the Athens Insomnia Scale (AIS), and the Chinese version of the Perceived Stress Scale (CPSS). DFI values were obtained from semen analyses. Least Absolute Shrinkage and Selection Operator (LASSO) regression was applied to identify potential predictors, followed by multivariable logistic regression to determine the final independent factors. A nomogram was developed and validated internally and externally. Model performance was evaluated using the area under the receiver operating characteristic curve (AUC), calibration curves, and the Hosmer–Lemeshow goodness-of-fit test.

**Results:**

Among the 746 participants, 237 (31.8%) exhibited abnormal DFI (>30%). Six independent predictors—age, body mass index (BMI), smoking, hot spring bathing, stress, and daily exercise duration—were identified as significant factors associated with abnormal DFI (all P< 0.05). The model showed excellent discrimination, with an AUC of 0.819 (95% CI: 0.771–0.867) in the training cohort and 0.814 (95% CI: 0.718–0.909) in the validation cohort. Calibration tests (Hosmer–Lemeshow P=0.798 and 0.817, respectively) indicated good model fit. In the external validation cohort, the AUC was 0.764 (95% CI: 0.707–0.821), suggesting satisfactory generalizability.

**Conclusion:**

A predictive model incorporating six modifiable lifestyle factors was developed and validated for assessing the risk of abnormal sperm DFI in infertile men. This nomogram provides a simple and clinically practical tool for early screening and individualized intervention to improve reproductive outcomes.

## 1 Introduction

Infertility has become an increasingly significant public health concern, affecting approximately 10%–15% of couples worldwide. Male factors alone account for about 30% of infertility cases, while combined male and female factors contribute to an additional 20% (1). In addition to conventional semen analysis, the sperm DNA fragmentation index (DFI) has emerged as an important biomarker for assessing sperm quality and male fertility potential (2). However, the association between DFI and assisted reproductive outcomes remains controversial. Some studies have reported that elevated DFI levels do not significantly influence the success rate of intracytoplasmic sperm injection (ICSI) (3), whereas others have demonstrated that DFI can predict pregnancy outcomes, embryo development potential, implantation rates, and the risk of genetic abnormalities (4, 5). Despite these inconsistencies, DFI is increasingly recognized as a valuable diagnostic indicator of male reproductive health. Lifestyle, defined as a pattern of daily behaviors and habits shaped by social context and personal values, encompasses multiple dimensions such as diet, exercise, sleep, stress, and environmental exposures (6). Increasing evidence indicates that lifestyle plays a crucial role in determining semen quality (7). Unhealthy behaviors, including smoking, physical inactivity, obesity, and chronic stress, can increase oxidative stress, leading to sperm DNA damage and impaired fertility potential. According to the World Health Organization Laboratory Manual for the Examination and Processing of Human Semen (6th Edition) (8), the clinical application of DFI testing is limited by cost and technical requirements, restricting its routine use in most healthcare settings. Therefore, identifying easily obtainable and noninvasive indicators that can predict abnormal DFI is of great clinical importance. In this context, the present study aimed to investigate the association between lifestyle factors and DFI in infertile men undergoing ICSI and to construct a predictive model based on these variables. By establishing and validating a nomogram, we sought to provide clinicians with a convenient and cost-effective tool for early identification of patients at risk of abnormal sperm DFI, thereby facilitating personalized counseling and preventive interventions.2.1.

## 2 Materials and methods

### 2.1 Study population and inclusion/exclusion criteria

This study included infertile men who underwent intracytoplasmic sperm injection and embryo transfer (ICSI-ET) treatment at the Reproductive Medicine Center of the Obstetrics and Gynecology Hospital affiliated with Tongji University between March 2023 and December 2023. ICSI was indicated for patients with severe male-factor infertility such as oligozoospermia, asthenozoospermia, teratozoospermia, or elevated sperm DFI. The decision to perform ICSI was based on established clinical criteria rather than DFI values alone, minimizing potential treatment bias associated with DFI levels.

Inclusion criteria were as follows: (1) meeting the diagnostic criteria for male infertility defined in the World Health Organization Laboratory Manual for the Examination and Processing of Human Semen (6th Edition)^8^ and undergoing ICSI-assisted conception; (2) normal male reproductive and physical examination results, without a history of conditions affecting sperm quality (e.g., reproductive tract infection, varicocele, or prostatitis); (3) no prior treatment or medication known to influence semen parameters before semen analysis; and (4) voluntary participation in the study.

Exclusion criteria included: (1) chromosomal abnormalities; (2) use of donor sperm, surgical sperm retrieval, or cryopreserved sperm for conception; (3) presence of severe chronic disease, malignancy, or other significant systemic conditions; (4) cases involving preimplantation genetic testing due to chromosomal or genetic abnormalities; (5) major life events within one month (e.g., bereavement, unemployment); and (6) unwillingness to complete the questionnaire.

### 2.2 Estimation method for sample size

To determine the sample size, we applied Riley’s sample size calculation method (9), using the formula:


$$n=\frac{100}{\left(1-R^{2}\right)\cdot P}$$

Previous surveys reported a detection rate of low DFI quality in Chinese infertile men of 43.19% (10), so we set P=0.43. The expected variance R^2^ was estimated using the empirical formula R^2^ = 2 x (AUC - 0.05). Based on the AUC value of 0.819 from the modeling group, treated as a pilot study, we calculated R^2^ = 0.671. This yielded a minimum sample size of 704 participants. Ultimately, 746 participants were enrolled in the study. All participants provided informed consent, and the study was conducted in accordance with the ethical principles of the Declaration of Helsinki. Ethical approval was obtained from the Ethics Committee of Tongji University Affiliated Obstetrics and Gynecology Hospital (Approval No.: KS2313).

### 2.3 Methods

#### 2.3.1 Survey instruments

1. General Demographic Data: A questionnaire was designed based on the Chinese Expert Consensus on Male Fertility Assessment (11), capturing the following variables: age, body mass index (BMI), household registration type, education level, employment status, exposure to high-risk occupational environments (e.g., high temperatures, radiation, or radioactive substances), smoking history (>20 cigarettes/day), alcohol consumption (40°–68°, >100 mL/day), habitual cola intake (>500 mL/day), habitual strong tea intake (>500 mL/day), habitual coffee intake (>500 mL/day), average daily exercise duration, average daily sleep duration, and sauna use (>once per week).
2. Athens Insomnia Scale (AIS) (12): Developed by Soldatos et al., this scale comprises eight items, each scored from 0 (none) to 3 (severe), yielding a total score ranging from 0 to 24. Higher scores indicate more severe insomnia. Scores of 0–3 suggest no sleep disorder, 4–6 indicate suspected insomnia, and >6 confirm insomnia. The scale demonstrates high reliability, with a Cronbach’s α coefficient of 0.880.
3. Chinese Version of the Perceived Stress Scale (CPSS) (12): Originally developed by Cohen et al. in 1983 and adapted by Yang Tingzhong et al. in 2003, this scale consists of 14 items across two dimensions, with a Cronbach’s α of 0.780, indicating strong structural validity. Total scores of 11–26 reflect low perceived stress, 27–41 indicate moderate stress, and >42 signify high stress levels.
4. Serum Total Testosterone Level: A 3 mL fasting venous blood sample was collected, and serum was separated and analyzed using chemiluminescence immunoassay to measure total testosterone levels.
5. DNA Fragmentation Index (DFI): Sperm chromatin structure assay (SCSA) was performed in accordance with the World Health Organization Laboratory Manual for the Examination and Processing of Human Semen (6th Edition) (8) to evaluate DFI.

#### 2.3.2 Data collection

With participants’ informed consent, researchers conducted a cross-sectional survey on the day their medical records were established, provided they met the inclusion and exclusion criteria. Participants were instructed to complete the questionnaire independently, reflecting their actual circumstances, in a private environment with ample time provided. All questionnaires were reviewed for completeness prior to collection. A total of 800 questionnaires were distributed, and 746 valid responses were collected, resulting in an effective response rate of 93.25%. DFI data were retrieved from semen analysis reports recorded in the Hospital Information System.

#### 2.3.3 Data classification

Previous studies suggest that in couples undergoing ICSI treatment, a sperm DFI >30% may exceed the DNA repair capacity of sperm and oocytes (13), potentially impacting IVF-ET outcomes. Accordingly, this study established a DFI threshold of >30% to indicate abnormal DFI. Participants with DFI >30% were assigned to the observation group, while those with DFI ≤30% were designated as the control group.

### 2.4 Statistical analysis

Continuous variables were expressed as mean ± standard deviation (SD) for normally distributed data or as median (interquartile range) for non-normally distributed data. Group comparisons for normally distributed continuous variables were conducted using the independent samples t-test. Categorical variables were reported as counts (percentages), with differences between groups analyzed using the χ² test. A p-value< 0.05 was considered statistically significant.

The study population was randomly divided into a modeling group and a validation group in an 8:2 ratio using a random number table method. Using data from the modeling group, LASSO regression analysis was performed with the “glmnet” package in R 4.2.1 to identify potential predictors. Multivariate logistic regression was then applied to determine the final predictors. A nomogram model for predicting DFI quality in infertile men was developed using the “rms” package in R 4.2.1. The discriminative ability of the nomogram was evaluated by plotting a receiver operating characteristic (ROC) curve based on the validation group data. Additionally, a calibration curve was generated to assess and refine the model’s performance, and the Hosmer-Lemeshow goodness-of-fit test was used to evaluate model accuracy.

## 3 Results

### 3.1 Comparison of clinical data on DFI status in infertile men

This study included 746 infertile male participants, of whom 237 had a DFI >30% and 509 had a DFI ≤30%, all meeting the inclusion criteria. Univariate analysis revealed significant differences between the two groups (p< 0.05) in age, BMI, smoking, alcohol consumption, daily exercise duration, exposure to high-risk occupational environments, sauna use, perceived stress, and insomnia. Detailed results are presented in **Table 1**.

**Table 1:** Univariate analysis of factors related to sperm fragmentation index in infertile male patients (n=746).

Item	Normal (*n*=509)	Abnormal (*n*=237)	*x^2^/t*	*P*
Age (year)			51.790	<0.001
20~29	72(14.15%)	23(9.70%)		
30~39	375(68.76%)	129(54.43%)		
39~49	87(17.09%)	70(29.54%)		
>49	0	15(6.33%)		
BMI (kg/m^2^)			28.457	<0.001
18.5~23.9	122 (23.97%)	24 (10.13%)		
23.9~27.9	287 (56.38%)	134 (56.54%)		
≥28.0	100 (19.65%)	79 (33.33%)		
Occupational Status			2.363	0.124
Non-employed	94 (18.47%)	33 (13.92%)		
Employed	415 (81.53%)	204 (86.08%)		
Account			1.675	0.196
Urban	479 (94.11%)	217 (91.56%)		
Agriculture	30 (5.89%)	20 (8.44%)		
Educational level			1.814	0.404
High school and below	157 (30.84%)	78 (32.91%)		
College/Undergraduate	266 (52.26%)	128 (54.01%)		
Master’s degree or above	86 (16.90%)	31 (13.08%)		
Smoking			43.831	<0.001
NO	438 (86.05%)	154 (64.98%)		
YES	71 (13.95%)	83 (35.02%)		
Drinking			39.821	<0.001
NO	446 (87.62%)	162 (68.35%)		
YES	63 (12.38%)	75 (31.65%)		
Cola			0.231	0.631
NO	148 (29.08%)	73 (30.80%)		
YES	361 (70.92%)	164 (69.20%)		
Tea			0.264	0.607
NO	212 (41.65%)	94 (39.66%)		
YES	297 (58.35%)	143 (60.34%)		
Coffee			1.093	0.296
NO	369 (72.49%)	163 (68.78%)		
YES	140 (27.51%)	74 (31.22%)		
Sleep Time(h)			5.596	0.133
<5	93 (18.27%)	41 (17.30%)		
5~7	267 (52.46%)	119 (50.21%)		
7~9	144 (28.29%)	69 (29.11%)		
>9	5 (0.98%)	8 (3.38%)		
Exercise Time(h)			52.248	<0.001
none	98 (20.51%)	94 (39.66%)		
0~0.5	115 (21.66%)	68 (28.69%)		
0.5~1	181 (35.48%)	41 (17.30%)		
>1	115 (22.35%)	34 (14.35%)		
High risk working environment			12.304	<0.001
NO	441 (86.64%)	181 (76.37%)		
YES	68 (13.36%)	56 (23.63%)		
Hot Spring			33.452	<0.001
NO	487 (95.68%)	197 (83.12%)		
YES	22 (4.32%)	40 (16.88%)		
Insomnia			29.255	<0.001
None	272 (53.44%)	83 (35.02%)		
Suspected Insomnia	154 (30.25%)	80 (33.76%)		
Insomnia	83 (16.31%)	74 (31.22%)		
Stress			19.369	<0.001
Low pressure	308 (60.51%)	103 (43.46%)		
Moderate pressure	135 (26.52%)	94 (39.66%)		
High pressure	66 (12.97%)	40 (16.88%)		
Testosterone	4.87 ± 1.46	4.67 ± 1.65	1.722	0.085

### 3.2 LASSO regression selection results

**Figure 1:**
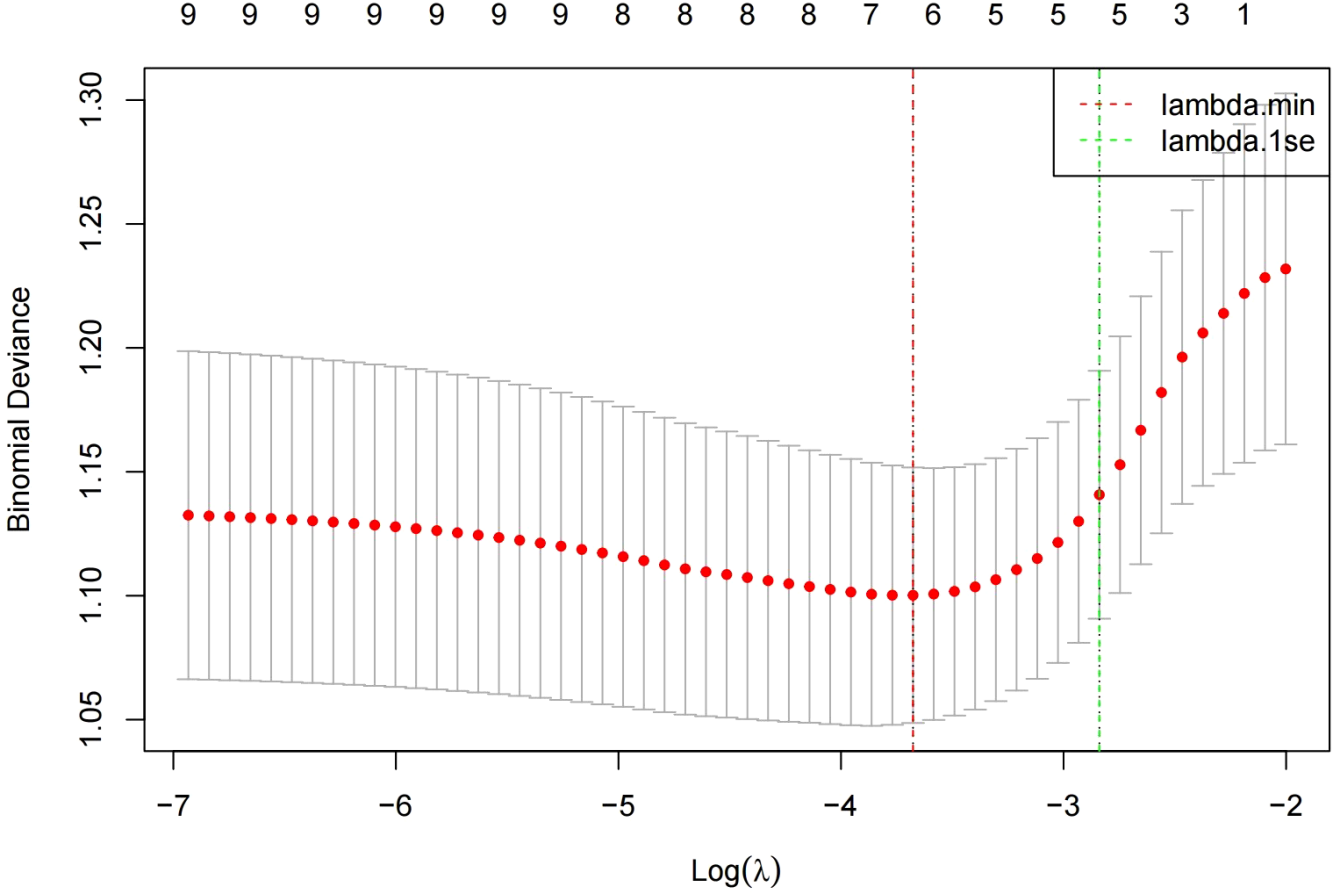
The dynamic process of variable screening in lasso regression.

**Figure 2:**
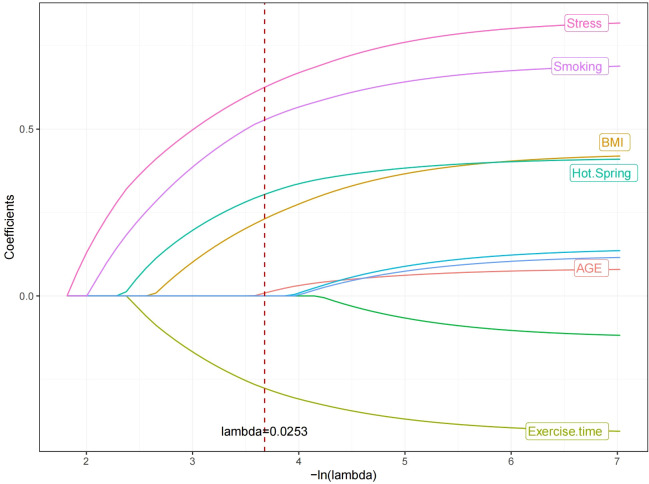
Cross-validation optimal parameter λ selection process.

In the modeling cohort, LASSO regression analysis determined the optimal penalty coefficient λ as 0.0253 through cross-validation, reducing the number of variables from nine to six potential predictors: Age, Smoking, BMI, hot spring, Stress and daily exercise time. See **Figures 1** and **2**.

### 3.3 Multivariate logistic regression analysis of factors affecting DFI in infertile men

Multivariate logistic regression was conducted to validate the variables identified through univariate analysis. Using abnormal DFI in infertile men as the dependent variable and the six factors selected via LASSO regression as independent variables, the analysis was performed. Variable assignments are detailed in **Table 2**. The results identified BMI, smoking, sauna use, age (40–49 years), daily exercise duration, and perceived stress as independent risk factors for DFI quality in infertile men (p< 0.05). Detailed results are presented in **Table 3**.

**Table 2:** Independent variable assignment.

Variable	Assignment
Outcome variable	
DFI	0= Normal, 1= Abnormal
Predictor	
BMI	0 = 18.5~23.9, 1 = 24~27.9, 2=≥28.0
Smoking	0=NO, 1=YES
Hot Spring	0=NO, 1=YES
Exercise Time (h)	0= None, 1 = 0~0.5, 2 = 0.5~1, 3=>1h
Age(year)	0 = 20~29, 1 = 30~39, 2 = 40~49, 3= >49
Stress	0= Low pressure, 1= Moderate pressure, 2= High pressure

**Table 3:** Multivariate logistic regression analysis of factors influencing DFI in infertile male patients (*n*=746).

Item	B	S.E.	Wald	P	OR	95%CI	
Constant	-2.091	0.401	27.203	<0.001	0.124	0.101~0.157	
BMI (based on 18.5-23.9 as a reference)			21.284	<0.001			
24~27.9	0.999	0.290	11.862	0.001	2.716	1.538~4.795	
≥28.0	1.458	0.316	21.254	<0.001	4.296	2.312~7.984	
Smoking (based on NO as a reference)	1.096	0.213	26.375	<0.001	2.993	1.970~4.547	
Hot Spring (based on NO as a reference)	1.415	0.309	20.925	0.001	4.115	2.245~7.544	
Exercise Time (based on NONE as a reference)			32.719	0.001			
0~0.5	-0.273	0.238	1.315	0.252	0.761	0.478~1.213	
0.5~1	-1.328	0.250	28.273	0.001	0.265	0.162~0.432	
>1h	-0.831	0.268	9.571	0.002	0.436	0.257~0.738	
Age (based on 20~29 as a reference)			9.294	0.026			
30~39	0.025	0.287	0.008	0.930	1.025	0.584~1.801	
40~49	0.656	0.319	4.227	0.040	1.928	1.031~3.604	
>49	21.999	9427.540	0.000	0.998	3.582E+9	0.000~	
Stress (based on Low pressure as a reference)			12.915	0.002			
Moderate pressure	0.646	0.201	10.282	0.001	1.908	1.285~2.831	
High pressure	0.663	0.259	27.203	0.010	1.940	1.169~3.221	

### 3.4 Construction of a nomogram model for DFI quality in infertile men

**Figure 3:**
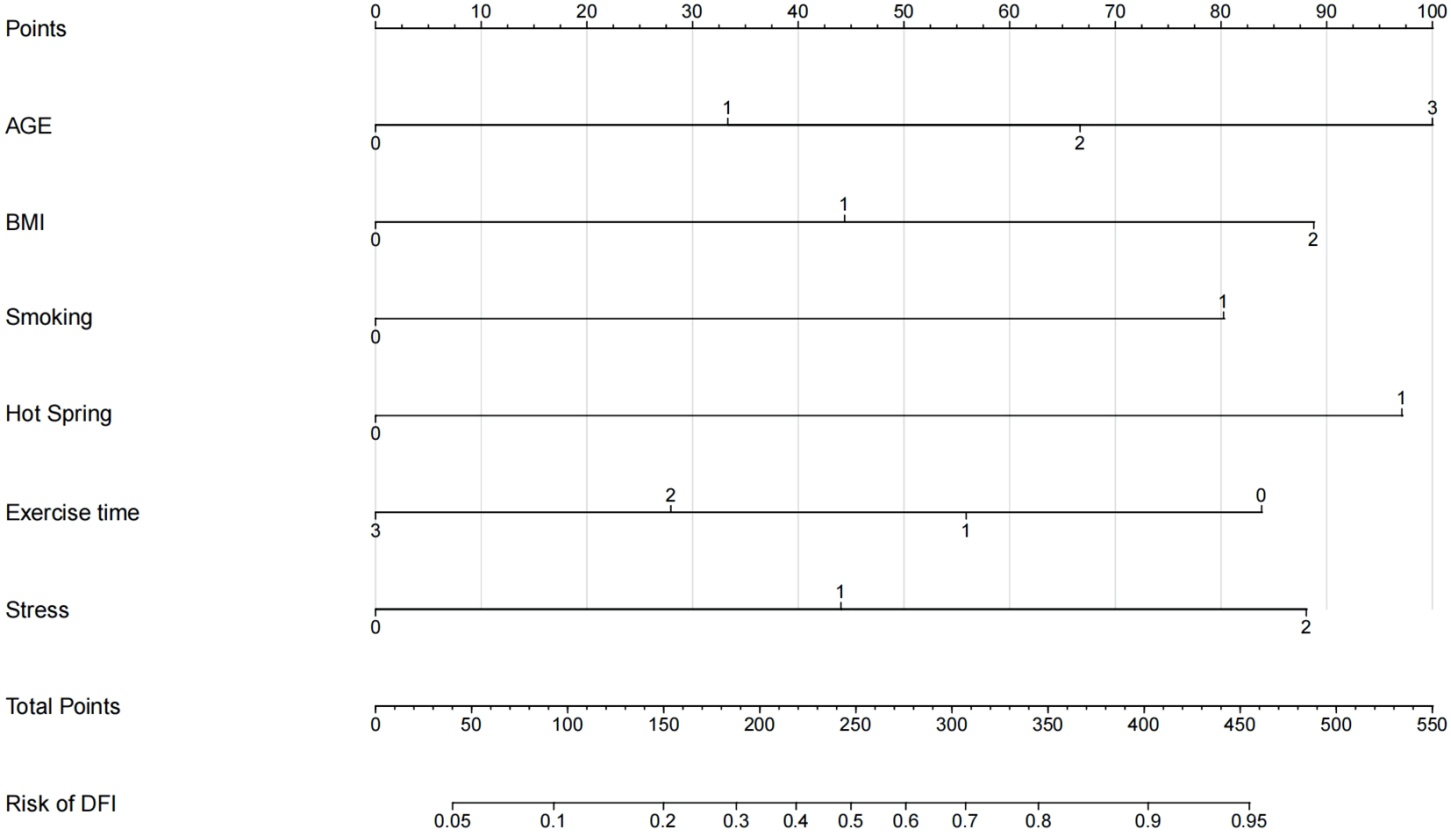
A column chart model for abnormal DFI quality in infertile men.

Using R 4.2.1, a nomogram model for predicting DFI quality in infertile men was developed based on multivariate logistic regression analysis, incorporating the six selected predictive factors. The nomogram’s total score ranges from 0 to 550 points, with the bottom axis indicating the probability of abnormal DFI. Higher total scores correspond to an increased risk of abnormal DFI. See **Figure 3** for details.

### 3.5 Validation of the nomogram model

**Figure 4:**
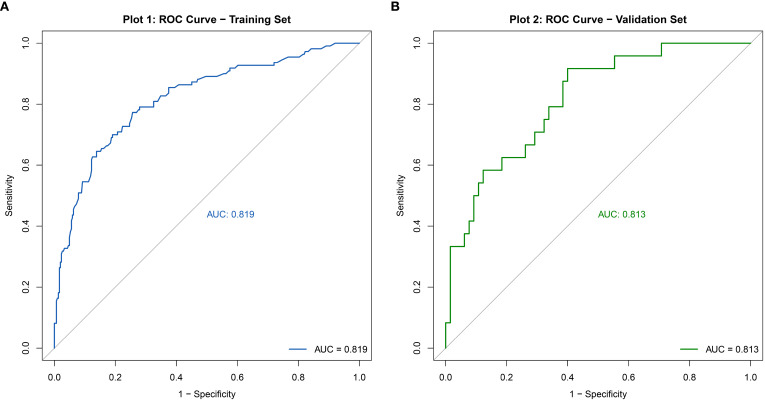
ROC curve of the nomogram model for predicting abnormal DFI quality in infertile men. **(A)** Training set. **(B)** Validation set.

**Figure 5:**
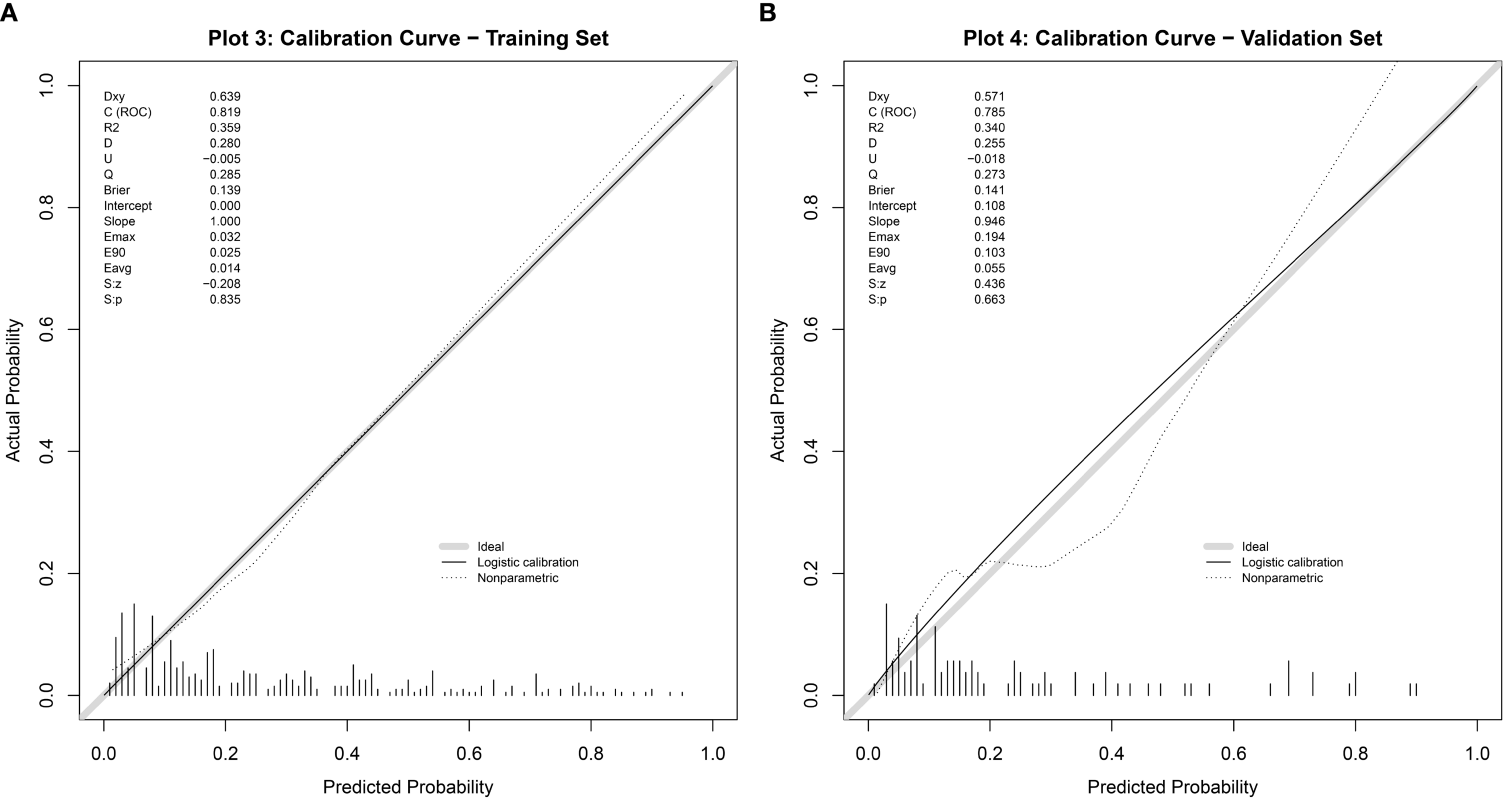
Calibration curve of the nomogram model for predicting abnormal DFI quality in infertile men. **(A)** Training set. **(B)** Validation set.

The discriminative ability of the nomogram model was evaluated by plotting receiver operating characteristic (ROC) curves. In the modeling set, the area under the curve (AUC) was 0.819 (95% CI: 0.771–0.867), with a sensitivity of 0.772 and specificity of 0.743. In the validation set, the AUC was 0.813 (95% CI: 0.718–0.909), with a sensitivity of 0.917 and specificity of 0.601 (see **Figure 4**). Calibration curves were generated, and the Hosmer-Lemeshow goodness-of-fit test was performed to assess model fit. In the modeling set, the test yielded a p-value of 0.798 and a Brier score of 0.139; in the validation set, the p-value was 0.817 with a Brier score of 0.148, indicating strong model consistency and good fit (see **Figure 5**).

### 3.6 External validation of column chart model

**Figure 6:**
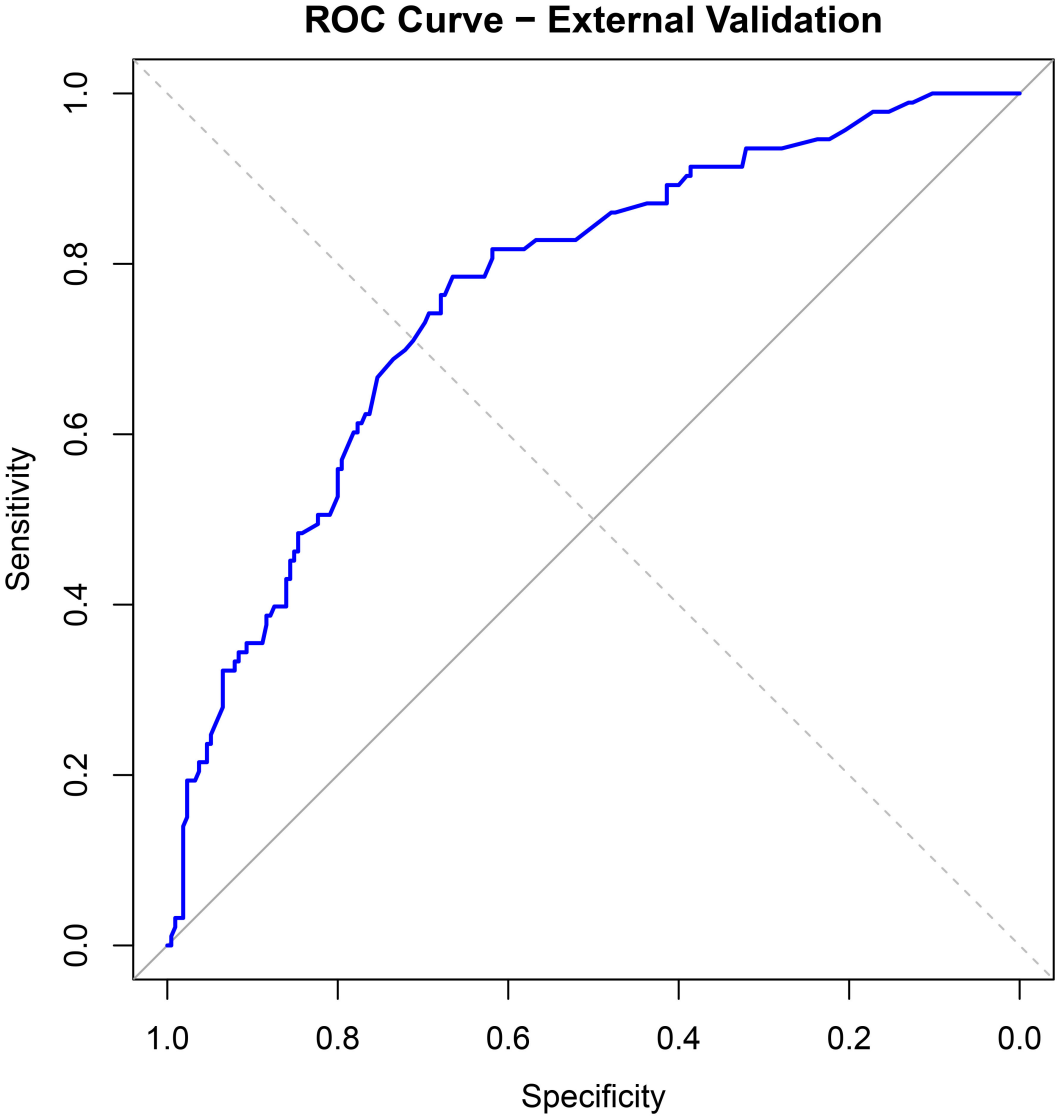
ROC curve for external validation of column chart model.

**Figure 7:**
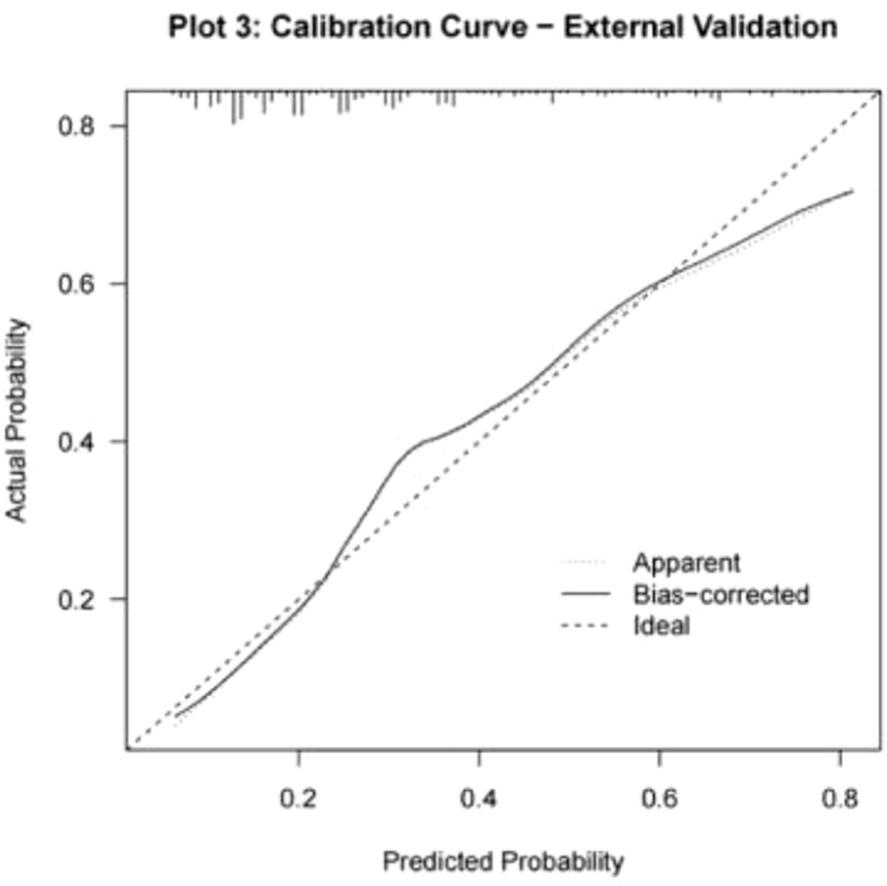
Calibration curve for external validation of column chart model.

For external validation, 308 infertile men who underwent ICSI-ET at the Reproductive Medicine Center of Xinhua Hospital, affiliated with Shanghai Jiao Tong University School of Medicine, from January to June 2024 were selected. Of these, 93 had abnormal DFI quality. The nomogram model developed in this study was applied for validation. ROC curve analysis yielded an AUC of 0.764 (95% CI: 0.707–0.821), with a sensitivity of 0.431 and specificity of 0.861 (see **Figure 6**). Calibration curves were generated, and the Hosmer-Lemeshow goodness-of-fit test was performed, resulting in a p-value of 0.938 and a Brier score of 0.177, indicating robust predictive performance in external validation (see **Figure 7**).

## 4 Discussion

The DNA fragmentation index (DFI) is a critical measure of sperm quality, directly reflecting sperm DNA integrity and serving as a key indicator in fertility assessments (14). Sperm DNA damage arises from a combination of irreversible factors, such as urinary tract infections, testicular injuries, chemotherapy, or radiotherapy, and modifiable lifestyle factors, including environmental exposures, diet, and psychological stress, which contribute to varying degrees of sperm DNA damage (15). As work patterns and environmental conditions evolve, lifestyle and mental stress among reproductive-age individuals have significantly changed, with poor lifestyle choices emerging as a leading threat to reproductive health globally and in China (16).

Our study investigated the impact of lifestyle factors on DFI in infertile men undergoing ICSI-assisted reproduction and developed a predictive nomogram model to identify high-risk factors early, supporting clinicians in implementing timely interventions and designing personalized, evidence-based fertility treatment plans to enhance sperm quality and fertility outcomes. Although the external validation AUC was slightly lower (0.764, 95% CI: 0.707–0.821) compared to the modeling set (0.819, 95% CI: 0.771–0.867; sensitivity: 0.772, specificity: 0.743, Brier score: 0.139) and validation set (0.813, 95% CI: 0.718–0.909; sensitivity: 0.917, specificity: 0.601, Brier score: 0.148), possibly due to differences in data distribution, limited sample size, or restricted feature variables, the model retained strong predictive ability and generalizability across populations. Higher specificity and lower Brier scores indicate a reduced likelihood of misdiagnosis. LASSO regression, ideal for high-dimensional datasets, was used to select relevant predictors and address multicollinearity (17), and combined with multivariate logistic regression, it identified six key risk factors—BMI, smoking, sauna use, age (40–49 years), daily exercise duration, and perceived stress—enabling the construction of a robust predictive model.

Our study aimed to investigate the impact of lifestyle factors on sperm DNA fragmentation index (DFI) in infertile men and develop a predictive nomogram model for risk assessment. A previous study in China developed a DFI prediction model based on traditional Chinese medicine syndromes and semen quality parameters, reporting an AUC of 0.745 (95% CI: 0.714–0.776) (18). While that study partially explored the predictive value of lifestyle factors on DFI, our research incorporated more specific lifestyle variables and achieved a higher AUC (0.819 for the modeling set, 95% CI: 0.771–0.867; 0.813 for the validation set, 95% CI: 0.718–0.909), indicating improved predictive performance. However, more comprehensive and precise data are needed to further identify risk factors associated with DFI and develop a more robust predictive model.

This study identified overweight/obesity, smoking, lack of exercise, sauna use, psychological stress, and advanced age as key contributors to abnormal sperm DNA fragmentation index (DFI) in infertile men, primarily through spermatogenic disorders and oxidative stress (19, 20). Oxidative stress, driven by excessive reactive oxygen species (ROS), activates apoptosis pathways, causing abnormal spermatogenesis in the testes and directly damaging sperm lipid membranes and DNA during transport, leading to elevated DFI (21, 22). High BMI, often linked to a high-calorie diet prevalent in this population, increases visceral fat, where aromatase converts testosterone to estradiol, suppressing the hypothalamic-pituitary-gonadal axis and reducing androgen levels, which impairs spermatogenesis and sperm maturation (23). Smoking elevates ROS in seminal plasma by increasing white blood cells or reducing antioxidant levels, resulting in DNA breaks (24). Regular exercise promotes testosterone production, but prolonged inactivity is associated with obesity, disrupting sperm DNA integrity (25), while intense exercise may decrease superoxide dismutase activity in testicular tissues and elevate inflammatory cytokines like interleukin-6, causing testicular damage, increased DFI, and higher ROS levels (26). Sauna use and similar activities raise testicular temperature, boosting ROS production and damaging sperm membranes, leading to testicular cell apoptosis and DFI fragmentation (27). Psychological stress disrupts hormone secretion and neurotransmitter metabolism, including monoamines and peptides, impairing the hypothalamic-pituitary-adrenal and gonadal axes, which elevates oxidative stress and inflammatory factors, further compromising DFI integrity (28). Additionally, advanced age was confirmed to negatively impact DFI quality, as cumulative unhealthy lifestyle effects exacerbate sperm nuclear immaturity, disrupt the protamine-to-histone ratio, impair chromatin packaging, and weaken DNA protection, increasing sperm DNA damage, consistent with prior research (29, 30).

Despite including a limited number of clinical variables, this study achieved favorable AUC values and demonstrated strong consistency between the training and validation sets, with the detection of these indicators being straightforward, easily obtainable, and feasible for implementation across hospitals of varying levels. Although the study focused on infertile men undergoing ICSI, the model’s applicability extends to the general male population, offering significant public health implications. Lifestyle profoundly influences reproductive health, and the core predictors identified in this model—BMI, smoking, lack of exercise, sauna use, stress, and advanced age—can serve as early warning indicators for reproductive health risks in men. Future research could apply this nomogram model in community health screenings to identify individuals at high risk for abnormal DFI and initiate preventive interventions to reduce infertility rates. While the model provides robust predictive value for early DFI screening in infertile men, its results should be used as an adjunct tool, with final diagnoses requiring confirmation through laboratory testing.

This study has several limitations. The cross-sectional design limits the ability to establish causality, and future research could employ longitudinal data with cross-lag models to better assess the directional impact of lifestyle factors on DFI. Additionally, the study focused on a limited set of risk factors, excluding environmental pollutants known to affect DFI, possibly due to constraints in sample size or statistical methods. Although the sample size was determined using Riley’s method, future studies should incorporate larger, multicenter, cross-regional samples and leverage advanced machine learning techniques to develop more accurate prediction models. Furthermore, as the study population comprised infertile men undergoing ICSI with a clear infertility diagnosis, the model’s predictive efficacy may be reduced or inapplicable for non-infertile males; future research should include broader male populations to thoroughly explore lifestyle impacts on sperm quality and develop dynamic classification models. Finally, incorporating embryo culture and pregnancy outcomes in future studies would provide more clinically relevant insights into the prognostic value of sperm DFI.

## 5 Conclusion

This study developed and validated an early predictive nomogram model for sperm DNA fragmentation index (DFI) quality in infertile men, based on six lifestyle factors, demonstrating significant predictive value and clinical applicability. Using LASSO regression and multivariate logistic regression, we identified BMI, smoking, sauna use, daily exercise duration, age (40–49 years), and psychological stress as independent risk factors for elevated DFI. By employing the nomogram derived from these factors, clinicians can directly estimate the probability of abnormal DFI, providing a robust tool for comprehensive semen quality assessment in infertile populations and guiding timely interventions to improve clinical outcomes and develop personalized treatment plans.

## Data Availability

The datasets used in this study are not publicly available due to institutional restrictions and privacy concerns. Requests to access the datasets should be directed to wangkeyfy@126.com.
